# Delivery of Antibiotics from Cementless Titanium-Alloy Cubes May Be a Novel Way to Control Postoperative Infections

**DOI:** 10.1155/2015/856859

**Published:** 2015-03-10

**Authors:** Martin B. Bezuidenhout, Anton D. van Staden, Gert A. Oosthuizen, Dimitar M. Dimitrov, Leon M. T. Dicks

**Affiliations:** ^1^Department of Industrial Engineering, University of Stellenbosch, Stellenbosch 7600, South Africa; ^2^Department of Microbiology, University of Stellenbosch, Stellenbosch 7600, South Africa

## Abstract

Bacterial colonisation and biofilm formation onto orthopaedic devices are difficult to eradicate. In most cases infection is treated by surgical removal of the implant and cleaning of the infected area, followed by extensive treatment with broad-spectrum antibiotics. Such treatment causes great discomfort, is expensive, and is not always successful. In this study we report on the release of vancomycin through polyethersulfone membranes from channels in cementless titanium-alloy cubes. The cubes were constructed with LaserCUSING from Ti6Al4V ELI powder. Vancomycin was released by non-Fickian anomalous (constraint) diffusion. Approximately 50% of the vancomycin was released within the first 17 h. However, sustained delivery of vancomycin for 100 h was possible by reinjecting the channels. Refillable implants may be a novel way to control postoperative infections.

## 1. Introduction

Bacterial infection is one of the greatest challenges in orthopaedic surgery [1–3]. Although the infection rate reported for total hip replacement (THR) surgery is less than 1%, the number of patients that are receiving implants is increasing [4]. This is also the case in total hip arthroplasty (THA) [1, 2, 5]. In most cases, infections associated with revision surgeries are caused by* Staphylococcus aureus* that forms a biofilm on the surface of the implants [6, 7]. The biofilm protects the bacteria against antibiotics and the host's immune system [8–10], which provides an additional challenge in the treatment of infections. Infection is usually controlled by removing the implant and extensive treatment of the infected site with broad-spectrum antibiotics [3, 11].

In several studies, the surfaces of implants were coated with an antimicrobial layer, or antimicrobial compounds were incorporated in the implants [12]. Customised implants were produced by using direct metal laser sintering (DMLS), selective laser melting (SLM), and electron beam melting (EBM) in metal additive manufacturing (AM) (Table 1). Femoral stems that are used in THR are largely manufactured from wrought material, using subtractive processes [13, 14].

In most studies conducted on implants with antimicrobial features bacterial infection could be controlled, but only for a short time. One of the major problems was the rapid decline in the activity levels of the antimicrobial agent [12]. A decrease in antibiotic activity levels below minimal inhibitory concentration (MIC) may lead to the selection of strains resistant to the specific antibiotic [15]. The ideal is thus to develop an implant that would release antibiotics in a controlled manner and at concentrations above MIC levels for as long as possible. To achieve the desired rate at which the antibiotic diffuses from the implant into the plasma, the geometric features of the implant would have to be simple and easy to manufacture. Additive manufacturing with layer-by-layer deposition of metal is perhaps the best technique to use [16].

Drug release depends on the physicochemical properties of the solutes, the structural characteristics of the material, and the possible interactions between these factors [17]. The three major drug release mechanisms are Fickian-, constraint-, and zero-order diffusion [18]. In Fick's model, molecules migrate from a high concentration to regions of low concentration, with a magnitude proportional to the concentration gradient and in linear relationship with time [18]. This means the drug relaxation time (*t*_*r*_) has to be greater than the solvent diffusion time *t*_*d*_ [19]. The drug is thus released independently from its concentration. Release of a drug at a constant rate is defined as zero-order diffusion. This type of diffusion provides the best control in drug release, ensuring absolute control over plasma concentrations and the frequency at which the drug has to be administered. An implant with controlled-release delivery should thus dispense the drug at a predetermined rate over a certain period. If *t*_*r*_ ≈ *t*_*d*_, the drug is released anomalously [19] and thus it is not at a constant rate.

In this study, vancomycin and gentamicin were used as model antibiotics. Vancomycin was chosen due to its use in the treatment of infections caused by methicillin-resistant strains of* S. aureus *(MRSA). Palacos R+G bone cement, containing 12.5 mg/g gentamicin (Heraeus Medical GmbH, Wehrnheim, Germany), was chosen since it has FDA approval for revision surgeries. Channels in titanium-alloy cubes were filled with vancomycin and then sealed with a low-molecular-weight cut-off polyethersulfone membrane. In another experiment, the channels were filled with Palacos R+G bone cement. The mechanism of drug release across the polyethersulfone membranes was determined by applying the Korsmeyer-Peppas equation [20, 21] for release from a thin slab:

(1) $$\begin{array}{c} \frac{M_{t}}{M_{\infty }}=Kt^{n}, \end{array}$$

where *M*_*t*_ = the cumulative mass of the drug released at time *t* and *M*_*∞*_ = the cumulative mass released at infinity. *K* = the release rate constant (units *t* ^−*n*^), which takes into consideration the structural and geometrical characteristics of the drug and the polymer releasing the drug. *n* = the diffusion exponent that defines the mechanism of release in the obtained profile. If *n* ≤ 0.5, the drug is released freely from the titanium-alloy cubes [22]. If *n* > 0.5, but <1.0, drug release is anomalous and indicative of diffusion under constraint; that is, the pore sizes restrict the release of the molecules [22]. In the case of the latter, molecules migrate in a nonlinear fashion over time and are influenced by interactions between the liquid and the solid phase [22], for example, the polyethersulfone membrane. If *n* = 1.0, release is, according to a case-II diffusion, also known as zero-order release [22]. If *n* > 1.0, release is classified as a super case-II diffusion [22].

## 2. Materials and Methods

### 2.1. Design and Filling of Titanium-Alloy Cubes

**Figure 1:**
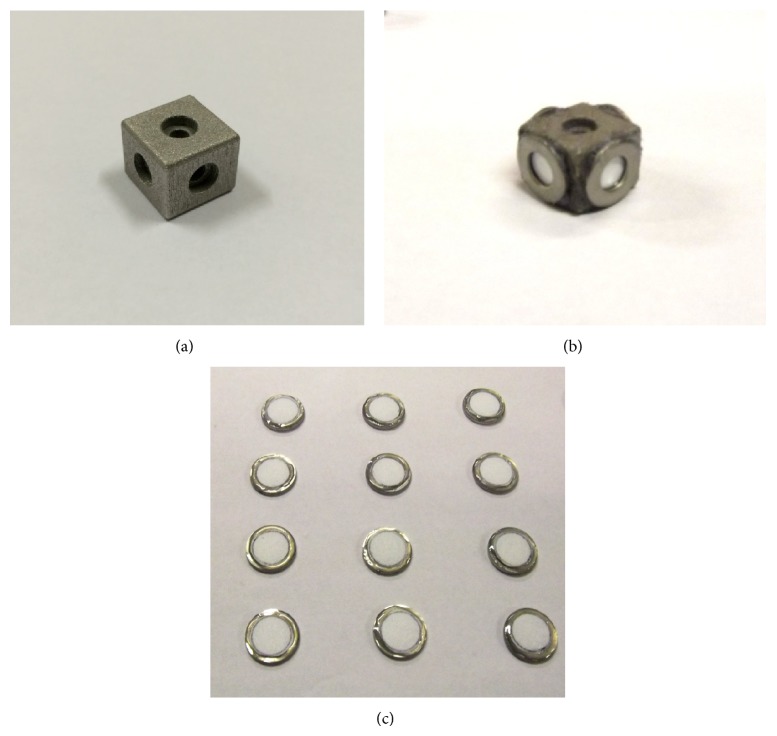
(a) Titanium-alloy cube with internal channels, (b) membranes fixed to vertical channel openings, and (c) membranes fixed to stainless steel washers.

Titanium-alloy cubes (15 × 15 × 12 mm, Figure 1) were manufactured from Ti6Al4V ELI powder (Concept Laser GmbH, Lichtenfels, Germany), with a particle size distribution of 38 to 55 *μ*m, on a M2 CUSING machine (Concept Laser GmbH). The channels were 3.5 mm in diameter and were centred perpendicular to the six surfaces (Figure 1(a)). The channel openings on the surface of the cube were 6 mm in diameter and 2 mm deep. The titanium-alloy cubes were autoclaved and cooled down to 25°C in a laminar flow cabinet.

Polyethersulfone membrane discs 8 mm in diameter, with a molecular cut-off of 5000 Da (YMMT 3000, Synder Filtration, Vacaville, CA), were placed over each of the four vertical channel openings and the edges glued to the titanium-alloy surface by applying a thin layer of waterproof epoxy adhesive (Figure 1(b)). The membranes were then prewetted for 6 h by injecting 60% (v/v) phosphate buffered saline (PBS, pH 7.4) with 40% (v/v) ethanol, refilling the reservoirs as needed to maintain continuous wetting. The ethanol lowered the surface tension of the membranes and prevented the formation of air bubbles. The membrane tension was kept by fixing a sterile stainless steel washer (6 mm inner diameter) over the membrane with epoxy adhesive (Figure 1(c)). The bottom opening of the vertical channel in the cube was sealed with commercially available antifungal clear silicone. Vancomycin HCl (Sigma Aldrich, St Louis, MO) was dissolved in sterile PBS to 2.5 mg/mL. The channels in each cube were filled with 400 *μ*L (1.0 mg) vancomycin. The open end of the vertical channel was covered with parafilm.

In a separate experiment, the titanium-alloy cubes were autoclaved and cooled in a laminar flow cabinet and the channels filled with Palacos R+G bone cement (ALBC), containing gentamicin. The Palacos R+G bone cement was prepared under atmospheric pressure in a monomer-to-polymer ratio of 1 mL to 2 g, according to the manufacturer's instructions. The two parts were mixed with a spatula for 30 sec and left for 60 sec to set. The cubes with ALBC were left to set for 30 min in a laminar flow cabinet with an ultraviolet light (254 nm wavelength). Protruding cement was filled off with a sterile file.

### 2.2. Testing of Antimicrobial Properties

Vancomycin-filled titanium-alloy cubes were placed in a closed beaker with 16 mL sterile PBS (pH 7.4) and placed on an orbital shaker (7 rpm) at 37°C. After 1, 3, 5, 8, 16, 24, and 43 h, the cubes were aseptically removed, 1 mL PBS was withdrawn, and stored in an Eppendorf tube at −20°C. The rest of the PBS was discarded and the beaker thoroughly rinsed with sterile PBS. The cubes were placed back in the beaker with a fresh solution of 16 mL sterile PBS. Sink conditions were used to ensure one-directional diffusion of vancomycin from the cubes into PBS. At two time points during the experiment (after 33 and 57 h), the vancomycin solution in each of the cubes was replaced with 400 *μ*L freshly prepared vancomycin. This was done to determine the effect of multiple doses.

At the end of the experiment the PBS samples were thawed, filtered, and transferred to glass autosampler vials (Chromacol, Herts, United Kingdom), using 17 mm diameter polyvinylidene fluoride (PVDF) disposable syringe filters with a pore size of 0.2 *μ*m (Chromacol). Vancomycin was detected by reverse-phase high performance liquid chromatography (RP-HPLC), based on the method specified in the vancomycin hydrochloride monograph of British Pharmacopoeia [28]. A Finnigan Surveyor Plus HPLC (Thermo Electron Corporation, Waltham, MA) and a Surveyor Plus pump coupled with an autosampler were used.

Twenty microliters of the sample was injected. A Surveyor UV/Vis Plus detector (set at a wavelength of 280 nm) was used, as described by British Pharmacopoeia [28]. The mobile phase consisted of HPLC-grade acetonitrile (AcN) (Merck) with 0.1% trifluoroacetic acid (TFA) and Milli-Q water (EMD Millipore) with 0.1% TFA. A C18 Thermo Scientific Hypersil GOLD reverse-phase chromatographic column (Thermo Fisher Scientific, Waltham, MA) of 100 mm × 4.6 mm and 5 *μ*m silica particle size was used. The gradients used are listed in Table 2. Preliminary runs revealed an average retention time of 4.67 ± 0.02 min for 14 injections.

Vancomycin HCl standards injected into the HPLC were 5.0, 12.5, 25.0, 50.0, 125.0, and 250.0 *μ*g/mL PBS. Integration of the detection peaks as well as linear regression was performed automatically by the software ChromQuest 4.2.34 version 3.1.6 (Thermo Electron Corporation). Linear regression was confirmed by manual calculations. Data recorded for the first 60% cumulative release values were fitted to the Korsmeyer-Peppas model. The data were evaluated using a two-tailed Student's *t*-test (*P* = 0.05) for independent samples with the assumption of equal variances. A null hypothesis indicated that the means are equal. Failure to reject the null hypothesis therefore statistically validates the fitted model to represent the same population as that of the recorded values. Simple linear regression was used to evaluate the linearity of the data when plotted against the linearised Korsmeyer-Peppas model.

Diffusion of gentamicin from the Palacos R+G ALBC was tested by monitoring the growth inhibition of* S. aureus*. The ALBC-filled cubes were placed on the surface of sterile Brain Heart Infusion (BHI) agar (Biolab Diagnostics, Biolab, Midrand, South Africa).* Staphylococcus aureus *strain Xen 36 and the methicillin-resistant strain Xen 31 (both from Caliper Life Sciences, Hopkinton, MA) were cultured separately in BHI broth (Biolab) at 37°C for 24 h to an optical density of 0.3 at 595 nm. This corresponded to log_10_ 6.7 ± 0.1 CFU/mL. Twenty microliters of each cell suspension was individually mixed with 20 mL melted BHI agar (Biolab), poured over the vancomycin-filled titanium-alloy cubes, and incubated at 37°C for 24 h. The medium was supplemented with 0.001% (w/v) cycloheximide (Sigma Aldrich) to prevent fungal growth. Images of the plates were taken; the cubes were aseptically removed, sterilised by wiping with 70% (v/v) ethanol (v/v), and left to dry in a sterile flow cabinet. The cubes were then transferred to a fresh plate with BHI agar and, as before, covered with* S. aureus* Xen 36 or Xen 31 imbedded in BHI agar. The plates were examined for growth inhibition after 24 h of incubation at 37°C. The cubes were removed and sterilised and the process was repeated until no zones of growth inhibition were observed. The surface area of the inhibition zones (excluding the area of the cube) was calculated and expressed as mm^2^.

## 3. Results and Discussion

**Figure 2:**
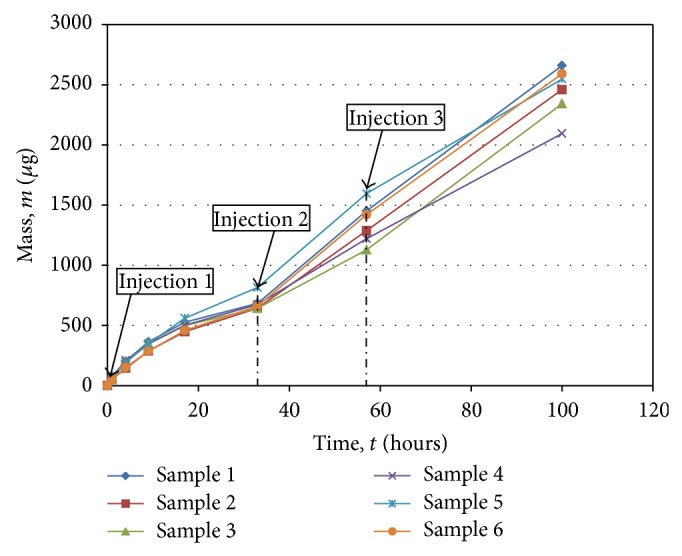
Cumulative mass of vancomycin released from titanium-alloy cubes. Values plotted are from six experiments.

Release of vancomycin from the cubes was without any initial burst (Figure 2). Lack of a burst release is characteristic of zero- or near zero-order diffusion [29]. Sustained near zero-order diffusion provides prolonged drug delivery as long as the drug remains stable.

To determine the values of *K* and *n*, the Korsmeyer-and-Peppas model was linearized:

(2) $$\begin{array}{c} \frac{M_{t}}{M_{\infty }}=Kt^{n}, \end{array}$$

(3) $$\begin{array}{c} \ln\left(\frac{M_{t}}{M_{\infty }}\right)=\ln K+\ln\left(t^{n}\right). \end{array}$$

**Figure 3:**
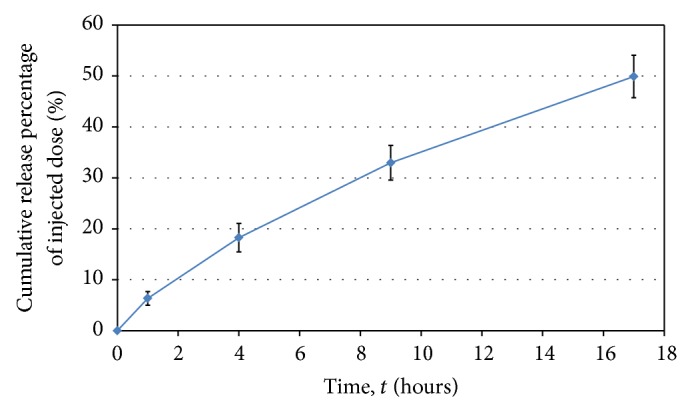
Cumulative percentage of vancomycin released from titanium-alloy cubes. Values are the average from six experiments. Standard deviations are shown.

A high degree of linearity (*R* ^2^ = 0.99) was obtained when the data were plotted using (3). The values obtained for *K* and *n* were 6.46 and 0.73, respectively. Since the *n*-value was higher than 0.5, but less than 1.0, the release of vancomycin is defined as non-Fickian and typical of that observed for anomalous (constraint) diffusion. Fitting of the data into the Korsmeyer-and-Peppas model at different time points is presented in Table 3 and is plotted in Figure 3.

An estimated 50.54% of vancomycin was released within the first 17 h (Figure 3) and falls within the 60% cut-off value for the diffusion approximation model developed by Korsmeyer-and-Peppas [19]. Sustained delivery of vancomycin over a longer period was possible by repeated filling of the cubes. From a practical point, the antibiotic levels in a titanium-alloy implant may be regulated by reinjection until infection is under control.

**Figure 4:**
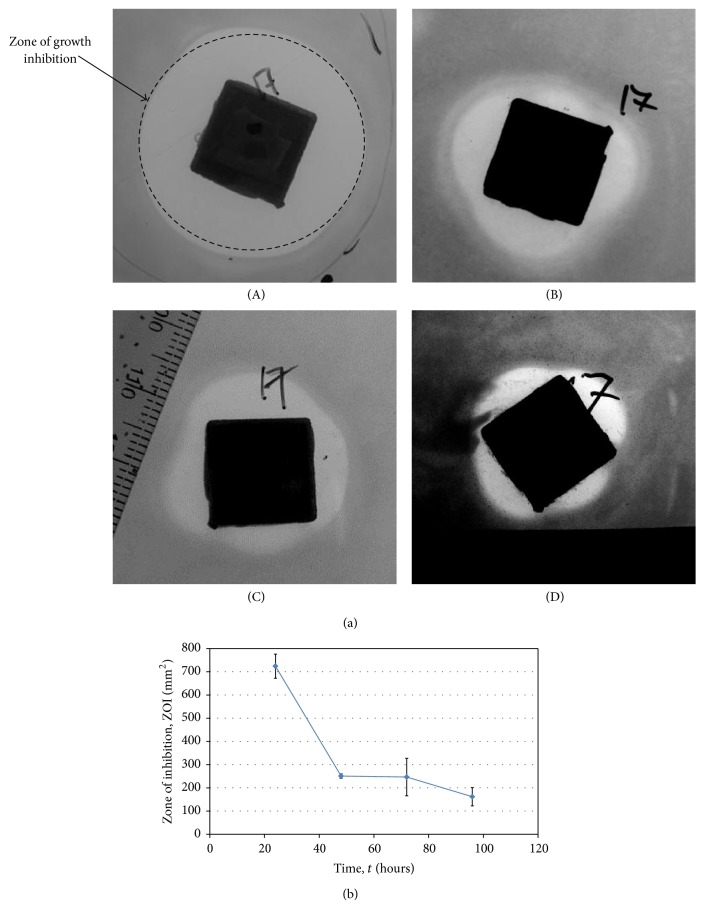
(a) Growth inhibition of* S. aureus* Xen 36 as gentamicin diffused from Palacos R+G ALBC-filled titanium-alloy cubes. Inhibition zones were recorded after 24 h (A), 48 h (B), 72 h (C), and 96 h (D). (b) Sizes of growth inhibition zones (in mm^2^) at each of these time points. Values are the average from six experiments. Standard deviations are shown.

Gentamicin diffused from Palacos R+G ALBC-filled cubes for 360 h, as recorded by growth inhibition of* S. aureus *Xen 36 (not shown). Clear zones of growth inhibition against strain Xen 36 were recorded for up to 96 h (Figure 4(a)). Most of the gentamicin was released after 24 h, as indicated by a large zone of growth inhibition (Figure 4(a)(A)), corresponding to a surface area of 725 mm^2^ (Figure 4(b)). The steady decline in growth inhibition zones over the next 24 h (Figures 4(a)(B) and 4(b)) indicated that gentamicin was released at a much slower rate. Zone sizes recorded at 72 and 96 h (Figures 4(a)(C) and 4(a)(D), resp.) ranged from 250 to 160 mm^2^ (Figure 4(b)), indicating that gentamicin was released at a more constant rate, but at less active levels. The high reduction in inhibition zone sizes after the first 24 h is typical of burst release. According to Poelstra et al. [29], 6-to-8 h after surgery is the most critical period to prevent bacterial infection. Based on the data presented here, diffusion of gentamicin from Palacos R+G into titanium-alloy cubes may control* S. aureus* infection for as long as 96 h.* Staphylococcus aureus* Xen 31 was less sensitive to gentamicin and growth inhibition was recorded for only the first 48 h (not shown). This corresponds to results published with cement discs [30]. As with most bacterial species, resistance to antibiotics is strain-specific [23]. This has to be taken into account in determining the treatment period of postoperative infections.

## 4. Conclusions

Diffusion of vancomycin and gentamicin from titanium-alloy cubes, prepared from LaserCUSING of Ti6Al4V ELI powder, indicated that it is possible to introduce the technology in implants and prevent secondary bacterial infections. Designing of an implant that allows repeatable filling with antibiotics may keep levels well above MIC for longer periods, thereby lowering the risk of strains developing resistance to antibiotics. The release of vancomycin through polyethersulfone membranes by constraint diffusion implies an interplay between Fickian diffusion and polymer relaxation. This research provides a basis for more detailed and specialised studies on developing a fully functional implant prototype. The technology may be extended to include other compounds such as anti-inflammatories. The invention could lead to a significant reduction in operating theatre time and medical costs.

## Figures and Tables

**Table 1:** Additive metal (AM) medical implants in clinical use.

Company	AM Type	Surgical application	Material	Year	Reference
Stanmore Implants Worldwide Ltd	DMLS^a^ and EBM^b^	Pelvic reconstruction	Ti-6Al-4V^d^	2010	[23]
Layerwise	SLM^c^	Facial reconstruction	Ti-6Al-4V	2012	[24]
Adler Ortho	EBM	Acetabular cups	Ti-6Al-4V	2007	[25]
Lima-Lto	EBM	Acetabular cups	Ti Grade 2	2007	[25]
Exactech	EBM	Acetabular cups	Ti-6Al-4V	2010	[25, 26]
Advanced Medical Technologies	EBM	Lumbar cage	Ti Grade 2	2009	[25, 27]

^a^Direct metal laser sintering.

^b^Electron beam melting.

^c^Selective laser melting.

^d^Titanium alloy.

**Table 2:** Mobile phase elution program used in RP-HPLC.

Time (min)	% Milli-Q (0.1% TFA)	% AcN (0.1% TFA)	Elution type
0-1	95	5	Isocratic
1–5	95–0	5–100	Linear gradient
5-6	0	100	Isocratic
6–11	0–95	100–5	Linear gradient
11-12	95	5	Isocratic

**Table 3:** Fitting of Korsmeyer-Peppas model to first 60% of cumulative drug release.

Time (hours)	% observed cumulative release	% estimated cumulative release	Square error	Sum of square errors	*P* value
1	6.34	6.46	0.01	2.01	0.97
4	18.26	17.67	0.35		
9	32.96	31.85	1.24		
17	49.90	50.54	0.42		
